# Valedictory Delivered at Commencement Exercises of the Ohio College of Dental Surgery, June 1, 1922

**Published:** 1922-06

**Authors:** Stanley L. N. Long

**Affiliations:** 1409 Elm Street, Cincinnati, O.


### VALEDICTORY DELIVERED AT COMMENCEMENT EXERCISES OF THE OHIO COLLEGE OF DENTAL SURGERY, JUNE 1, 1922

BY STANLEY L. N. LONG, D.D.S.

To the friends gathered here, to the professors who have been our guides, and to our fellow students and classmates, it is my privilege to give a last greeting and farewell.

The journey of life is along a road of many windings and turnings. Oftimes we find it extremely difficult to look either forward or backward—forward, because we can see only such a little way beyond our present position, backward, because of tears blinding our eyes—tears, perhaps, of sorrowful memories, but more often, it may be, of regret over lost past joys. As we walk the road, it seems long, this road of life; but when we glance back over the miles traveled, how pitiably short the road is, after all; while here and there, along its path, we see the gleam of a milestone marking the passing of one more mile.

Tonight, we as a class, reaching a milestone, pause to look back over the last four years with strange blending of regret and satisfaction. Ever since we began our studies, our eyes have turned to this hour as the goal of our ambition. We have studied for it, worked for it, planned for it, thought for it, dreamed for it, as the realization of our hopes and desires. As year after year passed, it seemed almost as far off as ever, and yet the bright star in our path led us on until at last we stand at the gateway, and half gladly, half sadly, look backward. For four years we have traveled hand in hand along the sheltered way, plucking blossoms of learning as they grew close at hand, and what is of even greater importance, gathering also the fruits of purity, nobility and truth that hereafter must be firmly ingrafted into every fiber of our natures. We have been carefully guarded by kind and zealous instructors from every adverse wind of thought, every taint of evil to be met in the world of action just beyond. Now our hands unclasp; sorrowfully we separate to go our different ways, to live the lives to which we have been called, no longer as a class, but as individuals. Is it strange, then, that we shrink from the parting word, and draw back into the shelter of this peaceful haven, as though fearful of the future?

Our school life has been a happy, inspiring life to each of us, a life of fellowship and fraternal intercourse that further cemented the class tie, and will, I trust, make us all look on each other in after years as something nearer and dearer than mere ordinary friends. Of course, we have had times of depression and anxiety. We have had sad thoughts too. Even from our freshman year we have realized that we were seeing our happiest days; that the parting hour only too soon would separate our paths for all times, making all our future widely different from our past. We have learned many lessons, some of them well. We realize that the most important lessons are not found in textbooks. As we step out of school life into life's school tonight, we are conscious that the hardest lessons are yet to come.

Gentlemen of the faculty—professors at whose feet we have so often sat, whose patience we have so often tried, whose best laid plans we have often foiled by our heedlessness, yet whose labors have been impressing, powerful influences on our lives—how can we voice our farewell to you? There has been the silent goodbye for many of you as we were together for the last time in your classroom. And in those last hours, the little commonplaces of question and answer, the quaint situations, the familiar twinkle of the eye, the handling of your illustrative objects, the endearing peculiarity of accent, all had a strange fascination for us. We dwelt on them as on the keepsakes of a departed friend and as we trend away, never more to be called upon, never more to listen, to laugh, to think with you and the boys, the goodbye which we voice to you now, then went from heart to heart. We thank you for the sacrifices you have made for us; to you has been given the task of impressing directly upon our minds those truths that shall develop the truest manhood of each nature, and of implanting in each brain and heart the germs of knowledge, whose perfect growth shall form lives of success, and whose fruitage be the crowning of well-spent lives. How well you have discharged the responsibility, the present but faintly shows, the future alone can tell how well, how faithfully you have labored in our behalf. We tremble as we leave you, for here we have relied upon your wisdom, your guidance; here we have sought counsel and assistance from you, who have ever been so able and willing to bestow it. Now we launch our little craft away, away from the shipyard, off the stocks, away from the master builder's hands. We go to battle with the waves, where there shall be none to guide or assist. Our own eyes must now watch the compass and scan the chart, our own hands must hold the rudder. Farewell kind, faithful teachers, farewell. If ever dark hours of defeat and failure come, bitterly will we rue the neglect with which we have met, alas, too many of your monitions. But when the banner waves high, and the glad shout of triumph echoes, we will think of you and say, that to you, to your wisdom and instructions, we know it all.

As we move off this stage our places are to be filled by others. We welcome you, fellow students, you seniors of next year. You are to enjoy the beneficent opportunities which we have enjoyed. May you improve them. You will fill the place which we now fill; may you fill it more worthily. We leave you, too, and extend the hand of parting. What more can we say than farewell, except to wish you well for the time to come. Together we have pursued our way through studious hours, we finish them just a little in advance of you, leaving you yet to linger a few months longer and then to follow us, giving place to those who will follow you. In all the possibilities of the future, in all that awaits you in the life to come, we bid you God-speed and fare you well.

Classmates, what a big thought it is that from this time on we have the shaping of our individual destinies in our own hands. All these years of our student life we have been for the most part, on the receiving hand. Life has been showering upon us its best gifts. While it is true that we have justly earned a certain portion of all that we have attained, there is a great deal of that subtle inner development, that gradual day-by-day character building, that almost invisible growth and expansion of the dormant man within us, for which we are still indebted to all the influences, both seen and unseen, that have been brought to bear upon all through all these years. We stand tonight at the very gateway of life's activities, prepared by all these years of careful, painstaking instruction and watchful, ever vigilant guidance, for the struggle with that real, vital existence that awaits us on the outer side. Think not that all is sunshine nor that fame and fortune will wait upon our bidding, "He who would win, must labor for the prize." If the thought arises are we adequate to the task of so shaping our course in life's dark maze as to reach the goal, the haven of success which we seek, let the success of others be our stimulus, and especially let the remembrance of the heights of fame and glory which so many of the graduates of our beloved alma mater have attained, spur us on in the constant battle of life. As we look back, how easy it is to estimate, by the land-marks along the road, the value received of our school career. Now the time has come for the working out of our "promise to pay." The world will at once commence to look to us to pay back into its treasury the wealth of good things it has for so long been bestowing upon us. It will demand our noblest revelations of character, our highest demonstrations of every latent possibility of attainment, our truest, tenderest attention to the needs of every brother and sister, our most faithful, self-sacrificing service. It will remind us, at every turn of the road, of that note always standing in our name with its never failing "for value received, I promise to pay." It will never once let us forget the cost of life, the constant expense that must be met, the unfailing price that must be paid for every gift, not in dollars and cents, but in service, in faithfulness to duty, in the uplift of our neighbor, yes, sometimes, perhaps, in unavailing pain and heart aches and tears. Classmates, is this a hard way to look at it? It should not be. It is only the small, immature mind that could take the narrow view of its significance. To us it should be a most inspiring thought, for it sets absolutely no level to the possibilities of our attainment. It is all to be just what we really want it to be. It is all to bring us just what we are willing to pay to gain what we desire. We can have what we will, for life itself will not say "Nay" when we come with the required fee in our hands, not as a beggar, but as a purchaser who knows what he wants and considers it worth the price demanded. So as we step forth through the gateway tonight, classmates, let us walk out into the world bravely, with a full realization of all that will be expected of us, but just as full a realization of our own ability to meet the requirements. There is absolutely nothing too expensive for us to buy with the assets at our disposal. Let us, then, resolve that we will keep our record so stainless, our ideals so lofty and unsullied, our account with life so accurately balanced, as we go, that there will never be any bad debts standing out against us, but that the "for value received, I promise to pay," as represented by our diplomas, may be only a pleasurable reminder of a most enjoyable duty and enviable privilege, as we look the world in the face, feeling that we have full claim upon such portions of it as we wish to make our own.

I can wish nothing higher or happier for us, than that through our lives, in joy and sorrow, in brightest sunshine and deepest shadow, there may remain with us the consciousness of a duty well performed, of suffering nobly endured, all of life faithfully lived. In the hope of such a future, with many pleasant memories of our fellowship and with the assurance of an unfailing affectionate remembrance, I bid you all goodbye.

1409 Elm Street, Cincinnati, O.

